# Experiences of bullying among nursing students during clinical practice: a scoping review of qualitative studies

**DOI:** 10.1186/s12912-024-02439-1

**Published:** 2024-11-14

**Authors:** Iyus Yosep, Nita Fitria, Ai Mardhiyah, Tuti Pahria, Ahmad Yamin, Rohman Hikmat

**Affiliations:** 1 Department of Mental Health, Faculty of Nursing, Universitas Padjadjaran, Jl. Raya Ir. Soekarno KM. 21, Hegarmanah, Jatinangor, Sumedang, Jawa Barat 45363 Indonesia; 2 Department of Fundamental Nursing, Faculty of Nursing, Universitas Padjadjaran, Sumedang, Jawa Barat Indonesia; 3 Department of Pediatric Nursing, Faculty of Nursing, Universitas Padjadjaran, Sumedang, Jawa Barat Indonesia; 4 Department of Medical Surgical Nursing, Faculty of Nursing, Universitas Padjadjaran, Sumedang, Jawa Barat Indonesia; 5 Department of Community Nursing, Faculty of Nursing, Universitas Padjadjaran, Sumedang, Jawa Barat Indonesia; 6 Master of Nursing Program, Faculty of Nursing, Universitas Padjadjaran, Sumedang, Jawa Barat Indonesia

**Keywords:** Bullying, Clinical practice, Nursing students

## Abstract

Bullying in the clinical setting has become a significant issue for nursing students. The experience of bullying during clinical practice can negatively affect students’ mental and physical health, as well as their professional development. Nursing students are often targets of verbal abuse, intimidation, and discrimination from various individuals in the clinical environment. This behavior not only harms individual students but also affects the quality of patient care. The aim of this study is to provide a deeper understanding of the phenomenon of bullying toward nursing students, identify research gaps, and offer recommendations for future research. This study employed a scoping review method, with articles sourced from four major databases: CINAHL, PubMed, Scopus, and Web of Science. The main keywords used included “bullying,” “nursing students,” “clinical practice,” and “verbal violence.” Inclusion criteria were studies with nurse participants, original research articles, and published within the last 10 years (2015–2024). Data were manually extracted using tables and analyzed through a qualitative descriptive approach. Eleven articles met the inclusion criteria. This scoping review focuses on the findings of an exploration of the experiences of bullying experienced by nursing students, identifies research gaps, and provides suggestions for future research. Findings showed that nursing students experience various forms of bullying, including verbal violence and intimidation, often triggered by power imbalances in interactions with clinical staff and mentors. The impact of bullying is not only detrimental to students’ mental health, but also affects their academic performance and quality of learning. Research gaps found include a lack of studies on the direct relationship between bullying and clinical assessment outcomes, as well as a lack of understanding of effective strategies to address bullying. This study recommends the need for clearer anti-bullying policies and transparent reporting systems, as well as further research to explore the impact of bullying in the context of nursing culture and education system.

## Introduction

Nursing education is important in producing competent and professional nursing staff. Nursing students are educated to become nurses who are able to provide high-quality nursing care and are dedicated to caring for patients [1]. However, nursing students often face the challenge of bullying during clinical practice. Bullying is defined as repeated and deliberate aggressive behavior to hurt or dominate other people, whether physically, verbally or psychologically [2]. In health education settings, bullying can occur between students, between students and clinical supervisors, or between students and hospital staff [3]. Bullying exerts a profound negative impact on nursing students, manifesting in elevated levels of stress, depression, and anxiety, as well as diminished motivation for learning and a subsequent decline in academic performance [4]. This can hinder the development of students’ competence and professionalism as prospective nurses [5].

The phenomenon of bullying against nursing students has become a global concern, with various studies showing an alarming prevalence throughout the world. In England, a survey revealed that more than 50% of nursing students reported experiencing bullying during clinical practice, both from colleagues, instructors and other health professionals [6]. In the United States, similar data shows that approximately 45% of nursing students experience harassment or bullying, which impacts their mental health and academic performance [7]. In South Korea, research found that 38.6% of nursing students experienced verbal bullying during clinical rotations, often caused by strict hierarchies in hospital settings [8].

The clinical practice environment is often a place where bullying against nursing students can occur, influenced by several factors. Strict hierarchies in hospitals or health institutions, where senior nurses and other health workers have greater authority, can create an imbalance of power that allows bullying behavior to occur [9]. High work pressure, especially in emergency situations or when the workload is excessive, can also increase stress among staff, who then channel their frustration onto students [10]. In addition, an unsupportive professional culture, where bullying behavior is ignored or even considered part of informal “education”, further exacerbates the situation [5]. On the other hand, nursing students are in a vulnerable position due to the difference in their level of experience and knowledge compared to more senior health workers [11]. They often feel isolated, dependent on guidance from senior nurses, and do not have the courage to report or challenge bullying behavior for fear of negative consequences for their assessment or future career opportunities [12].

Bullying in the clinical practice environment can have a significant impact on the education and careers of nursing students, both in the short and long term. Directly, bullying can cause excessive stress, anxiety, and decreased self-confidence, which interferes with their concentration and academic performance [13]. Students who are frequently victims of bullying may feel afraid to return to the clinic environment, experience sleep disturbances, and even show symptoms of depression [14]. The long-term impact of bullying is no less serious. Students who experience ongoing bullying are at risk of dropping out of the nursing education program because they feel unable to deal with the pressure [15]. Additionally, these negative experiences may reduce their interest in the nursing profession as a whole, resulting in a shortage of competent nurses in the future. Furthermore, students who continue to remain in the profession but with psychological scars from bullying experiences, may experience a decline in the quality of the health care they provide, due to loss of motivation, lower job satisfaction, and increased risk of burnout [16].

Although bullying of nursing students during clinical practice is an increasingly recognized issue in various countries, research specifically examining this experience is still very limited, especially in the context of nursing education [17]. Many studies have been conducted regarding bullying in the workplace or academic environments in general, but very few have focused on the unique experiences of nursing students in complex and stressful clinical environments [3, 6]. The lack of in-depth and systematic literature in this area creates a knowledge gap that hinders efforts to fully understand the scale of the problem and its impact. Therefore, a scoping review is needed to identify, analyze, and synthesize existing literature related to bullying in nursing students during clinical practice. This scoping review aims to provide a comprehensive overview of the available evidence, identify under-researched areas, and provide guidance for future research. The purpose of this study is to explore the bullying experiences of nursing students during clinical practice, found research gap and future research suggestions.

## Materials and methods

### Design

This study used a scoping review design with an approach developed by Arksey and O’Malley [18]. This design was chosen because the scoping review aims to comprehensively map the available literature and identify knowledge gaps in a particular topic. This approach is very suitable for the topic “Experiences of bullying among nursing students during clinical practice,” which has not yet been sufficiently explored. There were five main stages in Arksey and O’Malley’s approach: (1) Identifying research questions, (2) Identifying relevant studies, (3) Study selection, (4) Data extraction, and (5) Reporting of results. In this study, each stage was carried out systematically to ensure comprehensive coverage of the existing literature and consistency in the review process. The purpose of using this design was to provide a deeper understanding of the phenomenon of bullying toward nursing students, identify research gaps, and offer recommendations for future research.

### Search strategy and eligibility criteria

The search strategy was carried out in four main databases:: Scopus, PubMed, Web of Sciences, and CINAHL. The selection of this database was based on its completeness and credibility in providing health literature, especially in the fields of nursing and health education. Keywords used will include terms such as “bullying” “nursing students,” “clinical practice,” and “experiences,” using boolean operators to narrow the search. The keywords used are:

PubMed: (“Bullying“[MeSH Terms] OR “bullying” OR “harassment” OR “peer victimization” OR “intimidation”) AND (“Nursing Students“[MeSH Terms] OR “nursing students” OR “student nurses” OR” nurse students”) AND (“Clinical Practice“[MeSH Terms] OR “clinical practice” OR “clinical placement” OR “clinical education”) AND (“Experiences“[MeSH Terms] OR “experiences” OR “perceptions” OR “perspectives " OR “lived experiences”)

Scopus: (TITLE-ABS-KEY(“bullying” OR “harassment” OR “peer victimization” OR “intimidation”)) AND (TITLE-ABS-KEY(“nursing students” OR “student nurses” OR “nurse students”) ) AND (TITLE-ABS-KEY(“clinical practice” OR “clinical placement” OR “clinical education”)) AND (TITLE-ABS-KEY(“experiences” OR “perceptions” OR “perspectives” OR “lived experiences”) ).

CINAHL: (“bullying” OR “harassment” OR “peer victimization” OR “intimidation”) AND (“nursing students” OR “student nurses” OR “nurse students”) AND (“clinical practice” OR “clinical placement” OR” clinical education”) AND (“experiences” OR “perceptions” OR “perspectives” OR “lived experiences”).

Web of Sciences: TS=(“bullying” OR “harassment” OR “peer victimization” OR “intimidation”) AND TS=(“nursing students” OR “student nurses” OR “nurse students”) AND TS=(“clinical practice " OR “clinical placement” OR “clinical education”) AND TS=(“experiences” OR “perceptions” OR “perspectives” OR “lived experiences”).

**Fig. 1:**
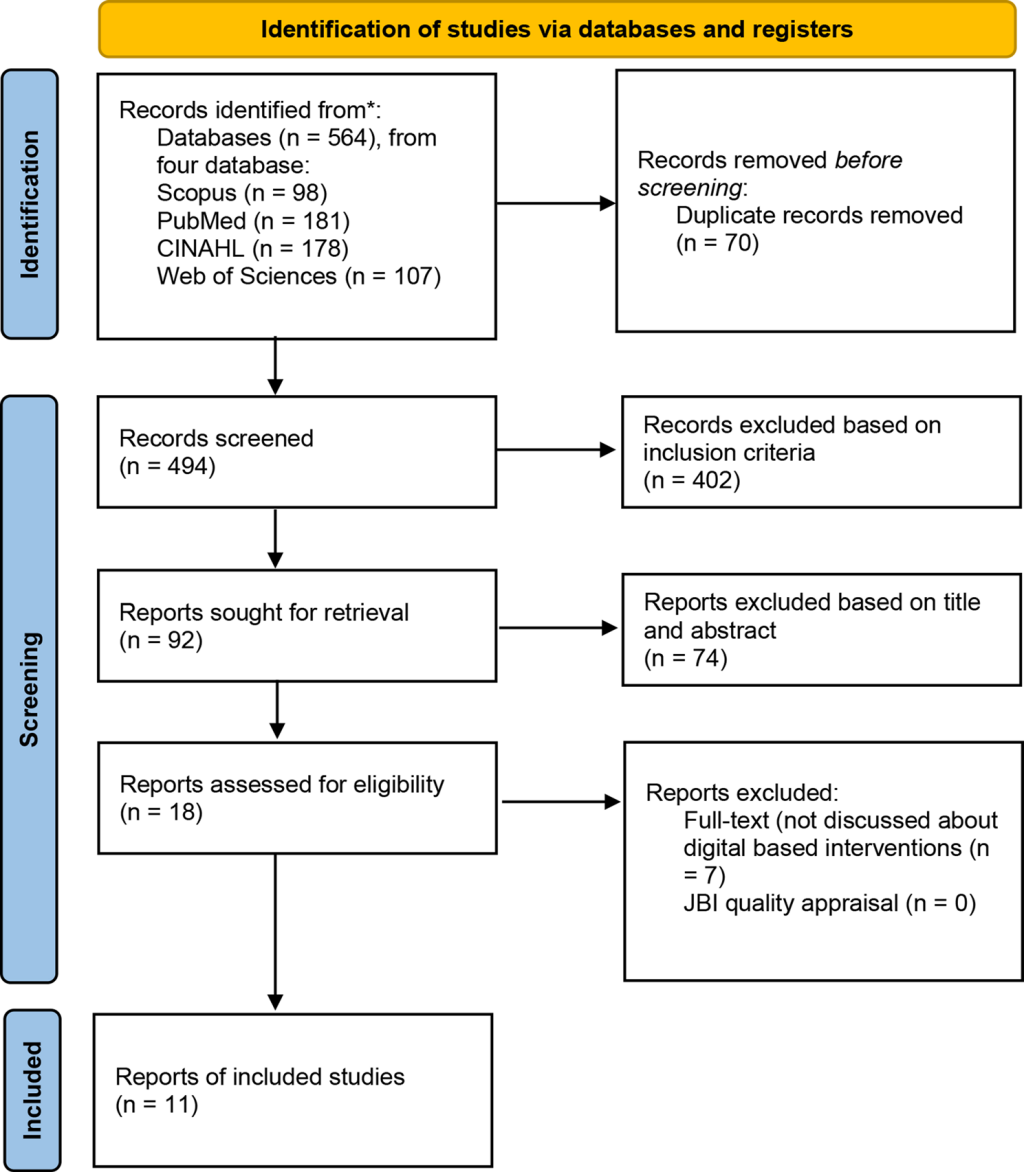
PRISMA Flow Diagram [19]

The research question to be answered is: “How do nursing students experience bullying during clinical practice?” An article search will be conducted using these keywords in each database, and the results will be reported using a PRISMA Flow Diagram to show the article screening process, from identification to final selection (Fig. 1).

### Inclusion and exclusion criteria

Inclusion and exclusion criteria were prepared based on the PCC (Population, Concept, Context) concept. The population in this study consisted of nursing students. The concept studied was the experience of bullying, while the context pertained to clinical practice in nursing education programs. The inclusion criteria encompassed studies that focused on the experiences of bullying among nursing students in various countries, original research, studies published in English, and those published within the last 10 years (2015–2024) to ensure that the information was current and could serve as a basis for recommendations. Conversely, the exclusion criteria encompassed studies that focused on bullying in non-clinical educational contexts, as well as articles that were solely opinion pieces or reviews. Reports in abstract form only and gray literature were excluded.

### Data extraction

Data was extracted manually using tables that included information such as authors, aim, study design, sample, country, instruments/questionnaires used, interventions, and results. Data extraction was carried out independently by two authors who were experts in their fields to ensure accuracy and reduce bias. If there was a difference of opinion between the two authors regarding the extracted data, a solution was sought through discussion. If differences persisted, a third author also an expert in the field, was invited to provide additional insights and help reach a consensus.

### Quality appraisal

Quality assessment of articles included in the scoping review was carried out using the JBI (Joanna Briggs Institute) quality appraisal tool [20]. The JBI quality appraisal method was utilized to assess the validity and reliability of various types of study designs, including qualitative studies. In the mixed methods design, the authors used the Mixed Methods Appraisal Tool (MMAT) to conduct the quality appraisal assessment [21]. This quality assessment was performed independently by two authors, with the standard for articles to be included in the review being a JBI score above 70%. If there was a difference of opinion between the two authors during the quality assessment, a discussion was held to reach a consensus. If consensus was not reached, a third author was invited to provide an additional assessment.

### Data analysis

Data analysis in this research was carried out descriptively and qualitatively using a thematic analysis approach. The analysis stage began with introducing the main themes derived from the research findings, which were then grouped based on their similarities and relevance. However, this process extended beyond mere clustering; it sought to uncover the underlying narratives that explained the phenomenon of bullying experienced by nursing students. In this regard, the analysis delved into various themes that emerged, such as the forms of bullying, the causes, and the impacts, exploring how these elements interacted to paint a comprehensive picture of the bullying phenomenon. Each theme was critically examined to identify its relationship with the research questions, emphasizing how these abstract concepts contributed to our understanding of the issue.

Two authors performed this analysis independently to ensure accurate interpretation. They analyzed data from 11 articles. If disagreements arose during the analysis process, a third author was invited to conduct additional analysis and help reach an agreed conclusion. This collaborative effort ensured a thorough evaluation of the themes, allowing for a deeper exploration of the narratives that connected the identified themes and illustrating the complexity of the bullying phenomenon in the nursing context. Ultimately, this approach not only ensured a comprehensive and balanced analysis but also significantly contributed to a more nuanced understanding of bullying in nursing education, aligning with the aims and questions of the research.

## Results

Based on the results of initial research, 564 reports were obtained from four databases, namely CINAHL, PubMed, Scopus, and Web of Sciences. Then, the authors carried out elimination based on duplicate articles using the Mendeley application, there were 70 duplicate articles. After that, the authors carried out elimination based on the inclusion criteria, there were 402 articles that did not match the inclusion criteria set by the authors. The authors read the titles and abstracts of the remaining articles, there were 18 suitable articles. Then, the authors read the articles in full text, and found 11 articles that discussed the bullying experiences of nursing students during clinical practice. The authors carried out a quality appraisal on articles that passed full text using JBI and MMAT assessment, all articles met the criteria with a score above 70%.

Based on the results of the 11 articles analyzed, there are various studies originating from several countries: China (1 article) [22], Ghana (1 article) [10], United Kingdom (2 articles) [23, 24], Hong Kong (1 article) [25], Iran (2 articles) [26, 27] Australia (1 article) [28], South Korea (2 articles) [29, 30], and New Zealand (1 article) [31]. The number of respondents in these studies ranged from 13 to 296 participants, with participant ages ranging from 20 to 25 years. All studies used a qualitative approach, with the majority of studies using qualitative descriptive methods or conventional content analysis. The research focus is varied, with most studies examining the experiences of bullying experienced by nursing students, both from colleagues and staff in the clinical environment. Some studies have also specifically explored verbal and sexual bullying, as well as the experience of reporting bullying incidents (Table 1). The authors found several themes in this research, namely the experience of bullying on nursing students and research gaps and future suggestions.

**Table 1:** Extraction data

No	Citation	Outcome	Country	Sample	Design	Type of bullying	Result
1.	(Qian et al., 2023)	Explores Chinese nursing students’ experiences of verbal violence in clinical practice and coping strategies.	China	21 nursing students in clinical practice ranged in age from 21 to 24, with average clinical practice duration of 7.09 months.	A descriptive qualitative study	Verbal bullying to staff	The study identified three main themes: verbal violence, its impact on students, and coping strategies. Nursing students experienced various forms of verbal abuse from patients, caregivers, peers, and supervisors, which negatively affected their health and well-being. Common coping strategies included seeking emotional support and forcing personal growth.
2.	(Amoo et al., 2021)	Describes bullying behaviors and effects on nursing students during clinical placements.	Ghana	30 students, 20 female Students who had experienced bullying were between the ages of 21 to 25 years	A qualitative phenomenological descriptive approach	Bullying Staff	Nursing students faced bullying in the form of shouting, isolation, humiliation, and being assigned tasks below their skill level. This led to a loss of confidence, stress, and anxiety.
3.	(Hallett et al., 2021)	Identifies prevalence and reporting of aggression among nursing students during clinical placements.	United Kingdom	36 students, 32 females, 5 focus groups Students who had experienced at least one clinical placement	Mixed methods Qualitative element: focus groups IPA	All Anyone	Focus groups highlighted issues such as widespread violence, racism, and bullying, along with the students’ lack of preparation and understanding.
4.	(Jack et al., 2021)	Examines nursing students’ experiences in reporting poor care.	United Kingdom	14 students, all female	Mixed methods (only qualitative element reported in this paper) Qualitative element: Semistructured interviews Constant comparison approach	Reporting	Four themes emerged: bullying, patient advocacy, lack of empathy, and poor care. Despite challenges, students were determined to advocate for patients, even when their concerns were initially dismissed.
5.	(Su et al., 2021)	Explores nursing students’ experiences in developing compassionate care skills.	Hongkong	22 participants, all female Final year nursing students	A descriptive qualitative study	All Anyone	Three positive themes included patient acceptance, professional growth, and emotional bonding. Negative themes included distrust, fear of violence, and heavy workloads. These findings suggest the need for a supportive practice environment and role models for compassionate care.
6.	(Mamaghani, 2018)	Details Iranian nursing students’ experiences in their clinical learning environment.	Iran	21, 12 female Undergraduate nursing students with average age of participants was 21 years.	qualitative study with conventional content analysis	Bullying Staff	Six categories were identified, including educational confusion, lack of evaluation procedures, limited opportunities, inappropriate staff interactions, a culture of bullying, and discrimination. These issues contributed to inadequate clinical education for nursing students.
7.	(Courtney-Pratt, 2018)	Investigates bullying experiences and coping strategies in clinical and academic settings.	Australia	29 first, second and third year undergraduate nursing students	Qualitative study with Directed content analysis	Bullying Anyone	Bullying occurred in both clinical and academic settings, perpetrated by various individuals. It caused anxiety, distress, and doubts about career choices. Students coped through avoidance, seeking support, and not formally reporting incidents.
8.	(Ahn & Choi, 2019)	Examines incivility experienced by nursing students during clinical practicum.	Korea	32 senior-year students were aged between 21 and 25 years, with an average age of 22.3 years	Qualitative content analysis	Bullying Staff	Students felt neglected and disrespected during clinical practicum, with unreasonable demands and a lack of role models. This highlights the need to improve clinical environments to reduce incivility.
9.	(Aliafsari Mamaghani et al., 2022)	Studies workplace violence against Iranian nursing students and its consequences.	Iran	20 Iranian nursing students aged between 20 and 22 years (mean (SD): 21.50	qualitative approach using conventional content analysis	Bullying Staff	Four categories were identified: vertical violence, horizontal violence, reactions to violence, and its consequences. Nurses were the primary perpetrators, using psychological and verbal abuse. Students responded in various ways, including reporting or disregarding the violence.
10	(Kim et al., 2018)	Describes nursing students’ experiences of sexual harassment during clinical practicum.	South Korea	13 nursing students	phenomenological qualitative approach	Sexual bullying Staff	Twelve themes emerged, such as unpreparedness, lack of education, power differentials, shame, and the long-term impact of harassment. Peer support was also highlighted.
11	(Minton & Birks, 2019)	Presents nursing students’ experiences of bullying during clinical placements.	New Zealand	296 students, 286 female 1st-3rd year	Mixed Methods Qualitative: Survey Coding and grouping into themes	Bullying Mostly staff	Students experienced various uncivil behaviors during clinical placements, resulting in physical, psychological, and financial consequences. Some students even considered leaving the nursing profession.

## Theme 1: experienced of bullying on nursing students

### Forms of bullying and violence

The forms of bullying and violence experienced by nursing students during clinical practice cover various dimensions, ranging from verbal violence to disrespectful behavior that damages the work environment. Multiform verbal violence is one of the most common forms of violence, where nursing students often become victims of patients, caregivers, colleagues, and even doctors [23]. Other bullying behaviors involve actions such as shouting, isolation, insults, and assigning tasks that are not within their competence [22]. Apart from that, violence in the form of ubiquity of violence such as racism and lack of empathy also often occurs, creating a culture that supports the practice of bullying culture [26]. In this environment, vertical and horizontal violence is prevalent, with students experiencing violence from senior staff and fellow colleagues [10]. Hostile behavior mean behavior often leads to rejection and hostile actions that damage the learning process [28]. Overall, these uncivil behaviors create an unsupportive clinical practice environment, hinder the professional development of nursing students, and affect the quality of the health services they provide [27].

### Impact of bullying

Bullying during clinical practice has a significant impact on nursing students, both immediately and long-term. Hurting and impacting, this bullying behavior damages their personal health and well-being, and has the potential to reduce workforce numbers in the nursing industry [10, 22]. The effects of bullying such as loss of self-confidence, stress and anxiety are common consequences experienced by students as a result of this bad treatment [24]. The consequences of violence include psychological and verbal violations that leave deep emotional wounds. Apart from that, bullying also has psychological and financial implications, where some students even consider leaving the nursing profession because of its detrimental impacts [26, 29]. This impact is not only limited to the study period, but also continues in long-term impacts, such as embarrassment, fear of further consequences, and negative impacts on the quality of patient care they provide in the future [29].

### Impact on education and clinical practice

The influence of bullying on the education and clinical practice of nursing students is very disturbing, with educational confusion being one of the main impacts. Students often feel confused due to the lack of systematic and consistent evaluation methods, which results in ambiguity in the learning and assessment process [22]. Apart from that, lack of respect and role models is a significant problem, where students feel unappreciated and do not get good role models from professionals in the clinical environment [23, 24]. The absence of evaluation procedures further exacerbates the situation, with the absence of clear and structured evaluation procedures in clinical education [31]. These limitations contribute to limited educational opportunities, where students do not get adequate educational opportunities to develop their clinical skills [25]. In the midst of these challenges, students must also face the dilemma of balancing self-preservation with obligations to patients, where they struggle to protect themselves from the impact of bullying while still fulfilling their responsibilities towards patient care [25]. This creates additional stress that interferes with their professional development and the quality of their learning during clinical practice.

### Strategies for dealing with bullying

When facing bullying during clinical practice, nursing students often use various struggling or coping strategies to survive. They tend to seek emotional support from others and force themselves to grow amidst stress [22]. Other coping strategies used include avoiding situations that trigger bullying, trying to survive by staying focused on educational goals, and seeking support from trusted academic staff, family and friends [31]. Reactions to student violence vary, ranging from fighting back or reporting incidents of violence, to ignoring them and considering them as normal things that happen in the clinical environment [26]. In the midst of these challenges, peer support plays an important role, where support from colleagues helps students feel stronger and able to face the bullying and violence situations they experience [29]. These strategies reflect students’ efforts to survive in a challenging environment, although they are not always effective in overcoming the root problem of bullying itself [27].

### Structural and systemic issues

Structural and systemic issues are key factors that exacerbate the experience of bullying among nursing students during clinical practice. The absence of systematic and consistent methods in clinical education and evaluation means that students do not receive clear guidance and adequate support, so they often feel trapped in uncontrolled situations [23, 30]. The significant power differential between students and clinic staff also exacerbates the issue, as students often feel they do not have the power or authority to fight back or respond effectively to bullying [24]. In addition, the lack of education and preparation to deal with bullying behavior makes students even more vulnerable, because they are not equipped with adequate skills or knowledge to face this challenge [27]. These structural and systemic issues create an unsupportive clinical environment, where bullying can flourish in the absence of effective intervention [30].

### Ethical and professional issues

Ethical and professional issues are a big challenge for nursing students who experience bullying during clinical practice. Student involvement in defending patient rights remains a priority, even though they have to face pressure and intimidation from the clinical environment [10]. However, fear of consequences or retaliation after reporting or facing bullying often prevents students from acting decisively, which impacts their sense of security and comfort in carrying out their duties [25]. The negative impact of bullying on the quality of patient care cannot be ignored; The stress and pressure experienced by students can reduce their ability to provide optimal care, thereby affecting patient well-being [26, 29]. This situation creates an ethical dilemma for students, where they must balance their professional obligations to patients with their own protection in the midst of an unsupportive environment [27].

## Theme 2: research gap and suggestions

Most existing studies focus on developed countries such as the UK, Australia, Hong Kong, South Korea, and New Zealand, as well as developing countries such as China, Ghana, and Iran. Research in developed countries generally focuses on the prevalence and impact of bullying in nursing education, but lacks in-depth exploration of effective long-term interventions to address this issue. On the other hand, research in developing countries tends to under-document institutional support and coping strategies that can help nursing students cope with bullying [10, 22]. However, there is a significant lack of research on bullying among nursing students in Southeast Asia, a region with highly diverse cultural characteristics and educational systems. Although countries in Southeast Asia face unique challenges in health education and power relations in the workplace, studies specifically exploring experiences of bullying in this region are limited.

Some studies identify coping strategies used by nursing students in the face of bullying, such as seeking emotional support and avoiding bullying situations [28]. For example, this study found that university students in Australia tended to avoid confrontation and seek support from peers, nursing students in South Korea used similar strategies to deal with disrespectful behavior in the clinical environment [29]. However, while these strategies appear to be helpful in the short term, no studies have in-depth evaluated their effectiveness on students’ long-term well-being. In addition, the institutional support available and how institutional interventions may strengthen or weaken the impact of these coping strategies is under-researched. This suggests a need for further research exploring the effectiveness of coping strategies and the role of institutional support in helping nursing students cope with bullying.

Several studies have shown significant challenges in reporting bullying by nursing students. Students often feel worried about the negative impact on their career if they report cases of bullying, the feelings of helplessness that students face in bullying situations [24, 32]. Nonetheless, in-depth research into the reporting mechanisms and resolution processes of bullying is lacking. Moreover, few have explored the impact of reporting on students who report bullying, both emotionally and professionally. Therefore, further research focusing on the reporting system, students’ experiences during the reporting process, as well as the effectiveness of the resolutions implemented is urgently needed.

Several studies have noted that the culture of violence and discrimination in the clinical environment has a significant impact on nursing students’ experiences. The culture of vertical and horizontal violence in clinical settings, which creates an unsupportive environment for students [26, 27]. However, more comprehensive research on how organizational culture and climate contribute to the prevalence and characteristics of bullying is still very limited. Deeper, focused research on the relationship between cultural factors, organizational climate, and bullying is needed to help understand how these elements influence interactions in the clinical environment. This exploration will be critical to creating strategies and policies that can build a more inclusive, safe, and supportive educational environment for nursing students.

## Discussion

The results of this study confirm that bullying of nursing students during clinical practice is a serious problem that affects their well-being and professional development. However, these findings need to be contextualized within a broader framework of theory and literature. The hierarchy of power theory in the context of nursing education, previous study explains that interactions between students and clinical staff, especially mentors or preceptors, are often hierarchically structured, which creates a power imbalance [24]. In this context, bullying can be viewed as a manifestation of power abuse, which not only hinders learning, but also creates anxiety and feelings of powerlessness in students.

From an educational perspective, particularly in the context of clinical assessment, bullying can affect objectivity and fairness in assessments made by mentors or staff nurses. Clinical assessments are often based on direct observations and interactions between students and clinical supervisors, which in some cases may not be free from personal biases due to the presence of bullying behaviors. For example, students who experienced bullying tended to feel demeaned and undervalued, which could impact their clinical performance [33]. This is relevant to the theory of affective bias in judgment, where judgments given by mentors or clinical staff may be influenced by personal interactions rather than based solely on the student’s objective performance [34].

Bullying can also disrupt the mentoring relationship, which should play an important role in supporting the development of students’ clinical skills. A healthy relationship between the student and mentor/preceptor is an important foundation for providing constructive feedback. However, when bullying occurs, this relationship becomes disrupted, so students may feel uncomfortable receiving or giving feedback openly [3]. This is in line with the research of Jackson et al. (2020), who found that bullying affected the quality of clinical learning and potentially reduced students’ academic performance. In addition, a research gap found in this study is the lack of research that explores the direct impact of bullying on clinical assessment outcomes and academic performance of nursing students. Previous studies have focused more on the psychological or emotional impact of bullying, such as stress, anxiety, and depression [9], but not many have examined how bullying specifically affects clinical judgment [34]. Therefore, further research is needed that explores the relationship between bullying and clinical judgment outcomes, as well as how these biases resulting from bullying may affect judgment decisions.

Several studies have identified that university students in countries such as Australia and South Korea tend to use coping strategies that involve avoiding bullying and seeking emotional support from peers or family [28, 29]. These strategies help in mitigating the immediate impact of bullying, however a significant weakness of these studies is the lack of in-depth evaluation of the long-term effectiveness of these strategies on students’ well-being. It is important to understand whether these coping strategies are temporary or can contribute to long-term well-being, including how students deal with stress in the clinical environment [35]. In addition, the role of institutional support in strengthening or weakening the impact of students’ coping strategies is often under-discussed in the literature. Strong support from the institution, such as clear anti-bullying policies and access to counseling services, could potentially improve students’ well-being, but this aspect still requires further research [36]. Therefore, more in-depth studies are needed to explore how institutions can play a more significant role in helping students deal with bullying.

In addition, the reporting mechanism and resolution process of bullying also face major challenges. Studies show that many students fear the negative impact on their careers if they report incidents of bullying, in addition to feeling powerless in dealing with the situation [24, 37]. This helplessness is often compounded by ineffective or non-transparent reporting systems, which discourage students from reporting their cases. This lack of research on reporting mechanisms and bullying resolution processes is an important shortcoming that must be addressed [11]. Further research is needed to identify how reporting systems can be improved, as well as to explore students’ experiences during the reporting process and the emotional and professional impact they feel afterwards. A better understanding of this will help institutions develop more effective policies and provide the necessary support for students who experience bullying.

This research revealed that nursing students face various forms of bullying and violence during clinical practice, including verbal violence from patients, caregivers, colleagues, and doctors, as well as intimidating behavior such as shouting, isolation, and assignment of tasks that are not in line with their competence. This phenomenon appears to be driven by a strong hierarchical culture in healthcare settings, where students’ relatively weak position in the power structure makes them vulnerable to these negative behaviors [15, 38]. Additionally, a lack of adequate training on how to deal with bullying in clinical settings exacerbates this situation, leaving students without adequate tools to address or report such experiences. These results are consistent with previous research findings, which show that bullying in clinical settings is often part of an organizational culture maintained by unwritten norms and a lack of firm action from management [39].

These findings underscore that bullying and violence in clinical practice is a systemic problem rooted in power dynamics and a lack of structural support. Vertical violence from senior staff and horizontal violence between fellow students also reflect significant competition and power imbalances in the clinic environment [40]. Additionally, the absence of systematic evaluation procedures and limited adequate educational opportunities further exacerbates confusion and dissatisfaction among students, ultimately contributing to increased levels of bullying [32, 41].

Additionally, some students choose to avoid direct confrontation or even ignore bullying incidents, strategies that reflect fear of further repercussions or an inability to confront bullies who are in positions of power. The study also found that some students reported incidents of violence, although these actions were rarely carried out due to fears of retaliation or stigma [15]. Compared with previous research, these reactions suggest similarities in coping patterns across nursing educational contexts, where support from peers and seeking protection from the broader academic community are considered important steps in maintaining professional integrity and personal well-being [12]. Many students still feel that bullying is an inevitable part of their journey, a view that requires more fundamental cultural change in the educational system and nursing practice [42].

The results of this study reveal that structural and systemic issues, such as the lack of systematic and consistent methods in clinical education and evaluation, affect nursing students’ ability to deal with bullying effectively. The absence of structured evaluation procedures causes confusion and uncertainty in clinical practice, which ultimately hinders the development of students’ professional skills [43]. This is also compounded by significant power differentials between students and clinic staff, which places students in a vulnerable position [14]. As revealed in previous studies, this power imbalance often makes students feel powerless to respond to bullying, so they tend to avoid conflict or choose to remain silent in order to maintain their position in the clinical environment [44].

On the other hand, ethical and professional issues are also a major concern, where nursing students are often caught in a dilemma between protecting patient rights and facing the risk of bullying. This study shows that although students engage in patient advocacy, fear of consequences or retribution from those in power makes them hesitant to report or confront bullying behavior [11]. This is in line with previous findings showing that bullying not only affects students’ well-being, but also negatively impacts the quality of patient care they provide [45]. Fear of potential harm if they stand up to bullying causes students to sacrifice quality care to avoid conflict, a phenomenon that shows how deeply bullying impacts nursing practice [46]. These issues highlight the need for structural reform in the nursing education system, including the development of clearer policies and stronger support for students in the face of bullying [46].

Future research should also explore more effective strategies to address bullying in the clinical practice setting. For example, interventions involving training for mentors/preceptors on the importance of a safe and bullying-free learning environment, as well as how to conduct objective assessments without being influenced by negative interpersonal interactions [47]. In addition, further research needs to explore more anonymous and safe reporting systems for students to report bullying without fear of negative impact on their assessment [3]. Meanwhile, in Southeast Asia, research on nursing student bullying is still very limited, despite the region’s highly diverse cultural characteristics and education systems. The strong hierarchical culture in many Southeast Asian countries, particularly in power relations in clinical settings, may also inhibit reporting of bullying, making it difficult to measure its prevalence and impact [48]. Research in this region is urgently needed to understand how local cultural norms influence the phenomenon of bullying and to design interventions that are appropriate to the local cultural context.

### Limitations

The limitations of this scoping review include several important aspects that need to be considered. First, the selection of articles from only four major databases (CINAHL, PubMed, Scopus, and Web of Science) may limit the scope of the results obtained, as it is possible that other relevant studies published in other databases or sources were not captured in this review. Secondly, this scoping review relied on qualitative descriptive methods in the analysis, which means that the interpretation of the results could be influenced by researcher bias. However, maximum efforts have been made to maintain objectivity by utilizing methodologies from the literature to enhance rigor and credibility, as well as validated tools for appraisal and data processing. These steps support efforts to reduce limitations. Finally, the limited number of articles that met the inclusion criteria may also affect the generalizability of the results, especially in understanding variations in bullying experiences across different clinical settings and cultures.

## Conclusion

This scoping review shows that bullying of nursing students during clinical practice is a serious issue that has a significant impact on their well-being and professional development. Findings suggest that bullying not only appears in the form of verbal violence from patients, coworkers, or clinical staff, but is also closely related to the power dynamics that exist within the nursing education environment. Strong hierarchies in interactions between students and clinical staff create power imbalances that facilitate bullying behaviors, which in turn affect clinical judgment, mentor relationships, and the quality of student learning and academic performance. The implications of these findings highlight the need for structural reforms in the nursing education system to effectively address the issue of bullying. Clear anti-bullying policies, training for mentors, and a transparent and safe reporting system should be introduced to support students in dealing with bullying behaviors. In addition, it is important for institutions to provide adequate emotional support for students and develop more effective recovery strategies.

Recommendations for future research include further exploration of the direct impact of bullying on nursing students’ clinical assessment outcomes and academic performance. More in-depth research is also needed to identify the relationship between bullying coping strategies and students’ long-term well-being. In addition, further studies on the reporting mechanism and resolution process of bullying should be conducted to understand students’ experiences and the emotional and professional impacts they experience. Research in the Southeast Asian region is also crucial to understand how local cultural norms influence the phenomenon of bullying and design interventions that are appropriate to the local cultural context.

## Data Availability

No datasets were generated or analysed during the current study.

## Abbreviations

JBI: Joanna Briggs Institute
